# Real-Time Environmental Monitoring for Aquaculture Using a LoRaWAN-Based IoT Sensor Network

**DOI:** 10.3390/s21237963

**Published:** 2021-11-29

**Authors:** Harvey Bates, Matthew Pierce, Allen Benter

**Affiliations:** NSW Department of Primary Industries, Orange Agricultural Institute, Orange, NSW 2800, Australia; matt.pierce@dpi.nsw.gov.au (M.P.); allen.benter@dpi.nsw.gov.au (A.B.)

**Keywords:** LoRaWAN, IoT, aquaculture, environment, monitoring

## Abstract

IoT-enabled devices are making it easier and cheaper than ever to capture in situ environmental data and deliver these data—in the form of graphical visualisations—to farmers in a matter of seconds. In this work we describe an aquaculture focused environmental monitoring network consisting of LoRaWAN-enabled atmospheric and marine sensors attached to buoys on Clyde River, located on the South Coast of New South Wales, Australia. This sensor network provides oyster farmers operating on the river with the capacity to make informed, accurate and rapid decisions that enhance their ability to respond to adverse environmental events—typically flooding and heat waves. The system represents an end-to-end approach that involves deploying a sensor network, analysing the data, creating visualisations in collaboration with farmers and delivering them to them in real-time via a website known as FarmDecisionTECH${}^{®}$. We compared this network with previously available infrastructure, the results of which demonstrate that an in situ weather station was ∼5 ${}^{\circ }$C hotter than the closest available real-time weather station (∼20 km away from Clyde River) during a summertime heat wave. Heat waves can result in oysters dying due to exposure if temperatures rise above 30 ${}^{\circ }$C for extended periods of time (such as heat waves), which will mean a loss in income for the farmers; thus, this work stresses the need for accurate in situ monitoring to prevent the loss of oysters through informed farm management practices. Finally, an approach is proposed to present high-dimensional datasets captured from the sensor network to oyster farmers in a clear and informative manner.

## 1. Introduction

Oysters are filter feeding bivalve molluscs that have hard calcified shells [1]. These organisms live in the intertidal and subtidal zones of many river systems, feeding on phytoplankton present in the upper water column where sunlight is available for photosynthesis [1,2]. Oysters are farmed globally, mainly for human consumption, and represent a significant proportion of global aquaculture production [3]. In Australia, the aquaculture industry is currently on the rise, and has an estimated net worth of $1.5 billion in 2021 [4]. This rise is primarily being driven by increasing consumer incomes and an increasing awareness on the positive effects of responsible aquaculture practices [3]. Oyster farming exists as one of the key components of Australia’s aquaculture industry, and within New South Wales (NSW) this industry accounts for 65% ($58.2 million) of total aquaculture production (in 2019/20) [4,5].

Oyster production provides valuable socio-economic and cultural benefits to local communities; however, as with any type of farming, oyster farming comes with inherent risks, such as disease outbreaks, pest invasion (e.g., mudworm—*Polydora websteri* or flatworm—*Imogine mcgrathi*) and negative environmental effects, such as flooding and heat waves [6,7,8,9]. These environmental factors have a significant effect on (1) the overall rate of oyster production, (2) the risk of food poisoning after consumption (e.g., norovirus) and (3) the risk that oysters may perish—especially younger oysters or vulnerable species [8,9,10]. As such, both farmers and consumers can suffer if appropriate management strategies are not in-place to prevent or minimise these risk factors.

The risk of mudworm or flatworm invasion can be contained by hanging oysters in the open air for several days [6,11]. However, this method is risky—especially in the summer months—as temperatures above 30 ${}^{\circ }$C can be fatal [6]. Farmers can use accurate local weather information to improve farm management. If high temperatures are detected early enough, farmers can cool down their oyster leases by spraying the oysters with seawater, or when using flip-baskets outside of intertidal zones, inverting their oyster baskets back into the water [6,12].

Heavy rainfall and subsequent flooding in the local or upper river system can have significant impacts on water quality. As oysters filter organic matter from the water column, they have the potential to ingest, and store for several days, bacteria (e.g., *Escherichia coli*), enteric viruses (Norovirus), heavy metals (e.g., lead, mercury, copper and zinc) and phytoplankton (e.g., cyanobactiera and diatoms) that pose a risk to human health [2,13,14]. These risks can be exacerbated when salinity in the water column decreases—as is the case after large rainfall events—and as such, the NSW Food Authority has implemented several regulations that halt oyster harvesting as a result of rainfall, sewage discharge, a phytoplankton bloom or low salinity [2,13]. Low salinity can have a significant effect on shellfish production as well, via reduced feeding and respiration rates as oysters shut their shells during these periods [15].

Real-time in situ data can provide significant benefits to the oyster industry as a means to enhance current farm management practices and provide early warnings for extreme weather events—such as heat waves and flooding. Additionally, these data could be used for future comparison under a changing climate. Real-time monitoring in agriculture and aquaculture is commonly employed to enhance farm management decisions [16,17]. However, the deployment of an environmental sensor network for monitoring *estuaries* presents several technical challenges that need to be addressed.

The first challenge to consider is the large sizes and properties of coastal estuaries that oyster leases typically occupy. As environmental conditions differ spatially, it is vital that multiple sensors are deployed to quantify the spatial variation in environmental variables, such as salinity [15]. However, this makes it difficult for real-time data to be captured remotely as commonly used cellular networks may not have sufficient signal across entire river systems (especially if the surrounding topography is varied). Alternatively, a low power wide area network (LPWAN) application, known as LoRaWAN, may be suitable for this function, as this form of communication enables the transfer of encrypted data over long distances (kilometres) while maintaining low-power consumption [18,19]. LoRaWAN requires a local receiving gateway to transfer the incoming packets to a network server—The Things Network (TTN) (The Things Industries, Amsterdam, NL)—to be decoded, after which a formatted (JSON) object (data) can be forwarded to a back-end database for storage and/or a front-end server for visualisation. LoRaWAN-enabled devices have previously been deployed in smart cities, within the agricultural sector and as an automated technique to monitor and control aquaculture ponds, amongst many other applications [20,21,22].

Secondly, as the sensor network is to be deployed in a saline environment, corrosion of sensitive electrical components is likely. For this reason, keeping the system as low-cost yet durable as possible allows for replacement parts to be acquired and fitted readily in the infrequent cases of failure. Typically LoRaWAN devices are low-cost compared to other Internet of Things (IoT)-enabled devices with similar functions, such as narrow-band IoT (NB-IoT), making LoRaWAN nodes cheap to replace if necessary [23]. Additionally, biofouling of submerged sensors also impacts the accuracy of environmental monitoring, as readings can drift overtime due to fouled sensor units [24]. This issue, however, can be managed with regular maintenance and preventive measures [24].

In this work, we present a real-time approach for monitoring environmental conditions on and within river systems using IoT-enabled systems and a LoRaWAN sensor network. This network was deployed in 2019 on Clyde River, located on the Southern coast of NSW, Australia, in collaboration with local oyster farmers as a means to increase their adoption of newly available digital technologies. The Clyde River represents roughly 11% (∼$6.1 million) of NSW’s total oyster production (in 2019–20) [5]. The sensor network consists of 10 buoys with submerged water quality sensors distributed from the head of the river at Batemans Bay, to the upper part of the estuary—8 km from Batemans Bay. Additionally, a local LoRaWAN-enabled automatic weather station (AWS) was deployed to provide an accurate representation of current weather conditions. The weather station is compared to currently available infrastructure to demonstrate the need for accurate in situ environmental monitoring systems in order for farmers to make informed decisions. While multiple works describing aquaculture monitoring using IoT devices exist, they often fail to apply the technology in field, or to denote its use by farmers [25]. Other works describe their use in the field but focus on the technological aspects, such as transmission performance [26,27].

The network produces a large amount of time-series and spatially resolved data which were automatically routed into an in-house developed data platform, known as FarmDecisionTECH${}^{®}$. This platform was used to create alerts and graphical visualisations for farmers that are accessible using both mobile and cellular devices. This work aims to present an end-to-end approach, by describing how the network was deployed, the challenges faced, the importance of in situ monitoring, how these data were analysed to make their interpretation accessible for farmers and how these data were delivered to farmers in real-time.

## 2. Methodology and Results

### 2.1. Location

**Figure 1:**
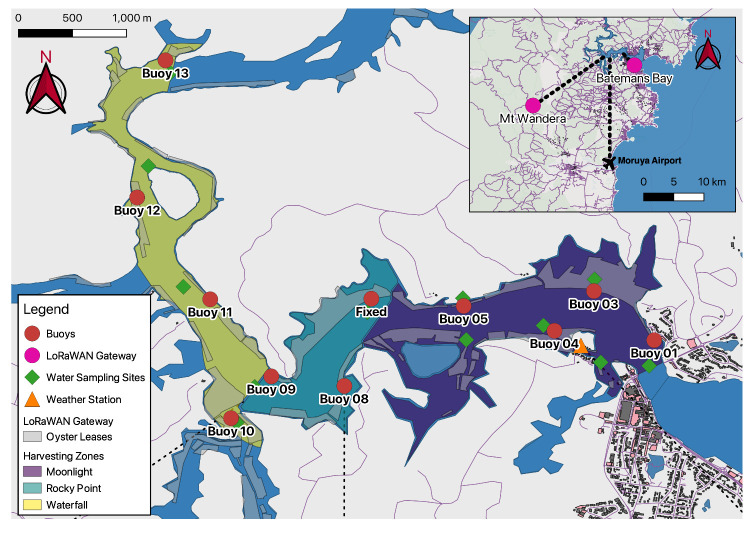
Locations of Clyde River and sensor network on the South Coast of NSW. Two maps of different scales are provided to represent the locations of deployed buoys (red circles) and other local developments, and the location of Batemans Bay in relation to Moruya Airport (black plane) and the regional LoRaWAN gateways (pink circles). Water sampling locations are represented as green diamonds, orange triangles represent weather stations, grey polygons define oyster leases and harvesting areas are indicated by purple (Moonlight), cyan (Rocky Point) and yellow (Waterfall) polygons.

The Clyde River, located on the Southern Coast of NSW, Australia (−35${}^{\circ }$42′30.3114″ 150${}^{\circ }$10′39.3486″), was chosen based upon its prominence as one of the largest oyster farming regions in NSW (Figure 1) [5]. Eurobodalla Shire Council, the local council surrounding Clyde River, had recently constructed a LoRaWAN-enabled network in consultation with Meshed (Meshed Pty Ltd, NSW, AUS), as a part of their Smart Cities Initiative. This infrastructure includes a LoRaWAN gateway located on a radio tower on-top of Mt. Wandera (∼20 km from Clyde River).

The river system is divided up into three main areas: (1) Waterfall, (2) Rocky Point and (3) Moonlight harvesting area (Figure 1). These harvesting areas are subject to different environmental requirements, as outlined by the NSW Food Authority (Table A1). Fixed buoys were positioned throughout these harvesting areas to coincide with water quality sampling sites and in relation to oyster production and/or vulnerable locations. The location of these buoys was subject to NSW Marine Parks Approval (permit number MEA19/172) and limited their location to areas outside Sanctuary Zones or amongst seagrass communities. Additionally, NSW Roads and Maritime were consulted to ensure the buoys’ positions were outside navigable waters and in conformity to the International Association of Marine Aids to Navigation and Lighthouse Authorities (IALA) (System A) special buoy requirements.

### 2.2. Hardware

**Figure 2:**
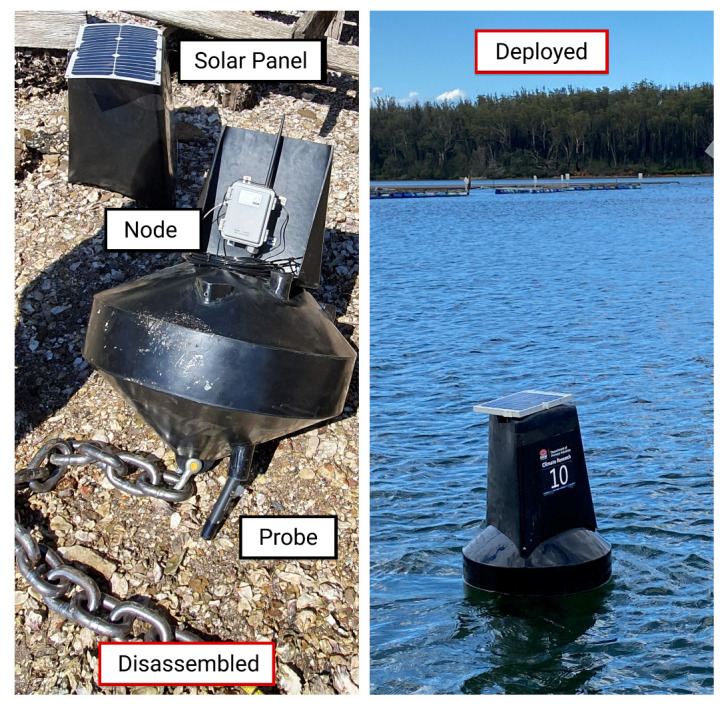
Black research buoy in a disassembled and deployed state on Clyde River. The left image shows a disassembled buoy with a solar panel to recharge a LoRaWAN sensor node. A salinity and temperature sensor (probe) sits underneath the buoy and communicates with the sensor node via a wired SDI-12 interface.

Black buoys (Data Buoy 600, Smart Buoy Co., Bairnsdale, Australia) were deployed based on IALA special buoy guidelines (Figure 2). These buoys were custom built to include a 37.5 mm tube, which extends through the buoy, for routing cables and underwater sensors. The buoys were secured to a ballast chain which extends from the base of the buoy followed by a rope to a 140 kg concrete anchor.

A LoRaWAN-enabled node (S-Node, ICT International, NSW, AUS) was attached to the top side of each of the buoys. This node features an IP65 enclosure to minimise the possibility of salt degrading electrical components and comes with a 12 V solar panel for maintaining battery charge. S-Nodes provide a SDI-12 interface for which we connected Ponsel C4E (AquaLabo, FRA) temperature and salinity sensors. These sensors were chosen based on the ease of use, as there are no moving parts, and therefore they can be cleaned easily. To reduce the rate of bio-fouling on the sensors, they were placed in 70 denier stockings (Big-W, NSW, AUS) and secured to the base of the buoy with cable-ties ∼40 cm below the surface of the water (representing the zone where oysters are situated).

Atmospheric weather conditions were assessed at fifteen-minute intervals using an ATMOS 41 automatic weather station (AWS) (Metergroup, NE, USA) connected to an S-Node. This AWS is capable of measuring a range of environmental variables without the use of moving parts such as wind vanes, making it robust enough for remote deployment (see Figure 1 for location). The AWS was situated on a pole away from buildings and trees, as specified by the AWS sitting guidelines provided by the Australian Bureau of Meteorology [28]. However, to represent the conditions the oysters were in, the AWS was placed over water four meters above the high-tide line.

Maintenance was carried out on the buoys and sensors at three-month intervals—as based on previous experience, this length of time was the sufficient to maintain reading accuracy and prevent destructive biofouling. This included scraping barnacles off the buoys, gently cleaning the sensors’ measuring heads with a brush or cloth and replacing stockings which covered the sensors. The AWS was maintained according to the same three-month interval, whereby the rainfall cone—a funnel that captures rainfall—had to be cleaned of debris which could block the flow of water through the device’s inbuilt rainfall sensor.

### 2.3. Software Flow

Incoming encrypted packets from TTN were automatically processed using a message queuing telemetry transport (MQTT) subscribe script written in bash. This script *subscribes* to the specific *topic* that each of the buoys are sending their data over and forwards the encrypted packets of data for storage (backup) and decoding using a combination of php and JavaScript. The decrypted data are then transferred to both ThingSpeak (MathWorks, MA, USA) and ThingsBoard (ThingsBoard, NY, USA) for redundancy measures.

**Figure 3:**
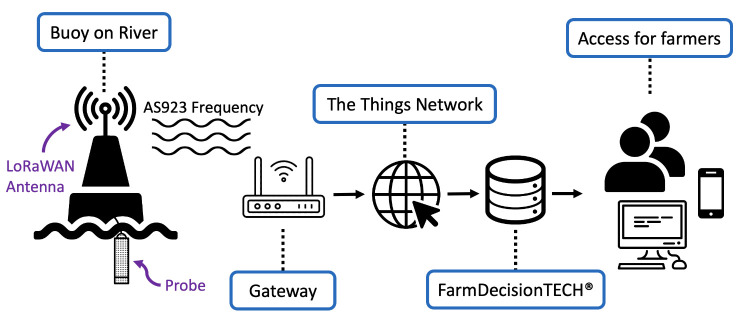
Flow of data from buoy to end-user. From the left, a buoy reads environmental variables from a submerged probe. These data are then transmitted over the AS923 frequency plan (from 921.8 to 923.4 MHz) to a local LoRaWAN gateway. The gateway forwards data over the Internet to TTN and into FarmDecisionTECH${}^{®}$. This website is then accessed by farmers and other end-users on mobile and desktop devices.

For ease of graphical visualisation, an application programming interface (API) was developed to operate on-top of the ThingsBoard API. This API is called through a WordPress (Automattic, SF, USA) site, known as FarmDecisionTECH${}^{®}$, and was plotted using ChartJS [29]. Oyster farmers can access this website using both mobile and desktop devices (Figure 3). The former is particularly important for oyster farmers, as they commonly check sensor readings while on the river and away from desktop computers. Based on view analytics on the FarmDecisionTECH${}^{®}$ website, ∼60% of users access the dashboard via their phone.

FarmDecisionTECH${}^{®}$ also provides the ability for farmers to create custom alerts when environmental readings (such as temperature or salinity) exceed custom thresholds. Additionally, oyster farmers were given private LoRaWAN temperature monitors for their oyster leases such that only they (or others with permissions) could access their data. This allows the oyster farmers to target oysters that are particularity vulnerable, such as young spats or sensitive species, while maintaining confidentiality on their locations.

### 2.4. LoRaWAN Performance

**Figure 4:**
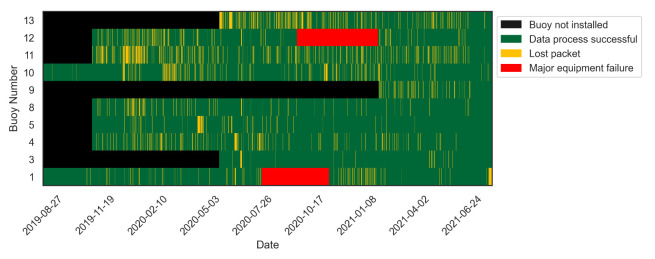
Operational information regarding each of the buoys across Clyde River. Black regions represent time before the buoy was installed, green represents data that were successfully sent and received; orange represents times when data were meant to be send and received but were not; and red indicates times when there was major equipment failure.

While LoRaWAN provides several benefits, this technology when applied to our application resulted in a number of *lost* packets of data. *Lost* data describes *uplinks* which were transmitted by a LoRaWAN node but not received by the LoRaWAN gateway. This can occur for several technical and environmental reasons and is often a result of poor received signal strength indicator (RSSI) values and signal-to-noise ratios (SNR) [30,31]. Figure 4 demonstrates the instances of packet loss and other downtime over the operating time of this sensor network. Buoys 1 and 10 were deployed first, followed by 4, 5, 8, 11 and 12, with Buoy 3 and 13 being deployed in mid 2020. Packet *loss* occurs somewhat randomly with the maximum amount of *lost* messages being ∼20% from Buoy 13. This buoy is surrounded by large hills and has no clear line of sight to any gateway resulting in low RSSI which could explain the poor message transmission. Although only ∼80% of data were received successfully, the randomness of this downtime allowed gaps in these data to be filled with different forms of interpolation. Two buoys suffered from long periods of downtime. Buoy 1 broke from its sea floor attachment during a large rainfall event in August of 2020, and Buoy 12 suffered from a sensor failure.

The LoRaWAN devices that are employed on Clyde River operate on different phases (i.e., the sampling time does not exactly match between buoys). This creates issues for data analysis, as environmental readings from multiple buoys must be processed by rounding each time stamp to the nearest hour. An hour was chosen, as on fifteen-minute sampling intervals each device is given four opportunities to successfully send at least one packet to a LoRaWAN gateway. If there were still missing data for a particular time stamp, an interpolation was applied to fill in any missing values.

### 2.5. Local Weather

**Figure 5:**
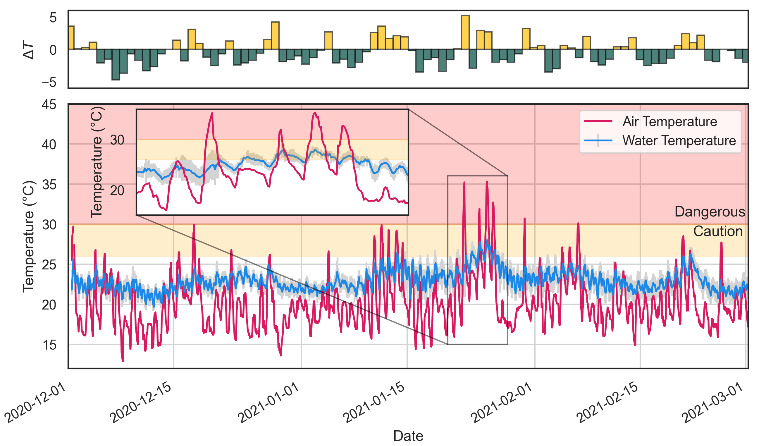
Air (pink line) and water (blue line) temperatures on Clyde River during the summer months (December 2020 to March 2021). Water temperature is the average of all ten buoys ± the standard deviation. Air temperature was captured using an AWS located on Budd Island. Background colours are used to represent temperatures above 26 ${}^{\circ }$C (orange) and above 30 ${}^{\circ }$C (red). The insert depicts a zoomed-in area of interest. Bar chart above main figure demonstrates the difference between (ΔT) the temperatures recorded at Moruya Airport and at Budd Island. Yellow indicates higher temperatures at Batemans Bay, green indicates lower.

As heat waves are detrimental to oysters’ survival, a local weather station was deployed to track extreme weather events in real-time [6]. Figure 5 shows both atmospheric (air) temperature and water temperatures on Clyde River during the summer months (December 2020–March 2021). This date range represents the period of time where oysters are most vulnerable to extreme weather events, such as heat waves. Over this three month period there were multiple occasions where air temperature entered a dangerous zone (≥30 ${}^{\circ }$C) where oyster mortality was possible if proper management practices were not implemented by oyster farmers [6]. Most notably, there was a seven day period (highlighted as an an insert in Figure 5) where the maximum daily air temperature exceeded 30 ${}^{\circ }$C. This period of high air temperature coincides with a rise in water temperature to a maximum reading of 27.99 ± 0.44  ${}^{\circ }$C.

Shown in the upper part of Figure 5 is a comparison between the *local* real-time Bureau of Meteorology (BoM) weather station located at Moruya Airport and the AWS deployed at Batemans Bay (see Figure 1 for location). These data represent the differences between the maximum temperature each day over the summer months at Batemans Bay ($T_{BB}$) and the maximum temperature at Moruya Airport ($T_{MYA}$) for the same day:

(1) $$\Delta T=max\left(T_{BB}\right)-max\left(T_{MYA}\right)$$

The data featured in Figure 5 indicate that the maximum temperature at Batemans Bay (i.e., where the oyster leases are located) can be ∼5 ${}^{\circ }$C higher than temperatures recorded at Mouyra Airport. There are BoM weather stations located closer to Batemans Bay, one at Nelligen (∼8 km from Batemans Bay) and another at Batemans Bay Catlina Country Club. However, these sites do not offer real-time data and therefore do not provide information for oyster farmers to make informed decisions with. Additionally, neither of the BoM weather stations reflected the intensity of the heat waves featured in Figure 5.

### 2.6. Harvesting

Regulations overseen by NSW Food Authority dictate that salinity in each of the harvesting areas must exceed a set threshold before harvest area openings can be assessed (Table A1). This threshold is harvest area-specific. Moonlight and Rocky Point require salinity readings ≥22 g/kg. Waterfall, however, requires ≥23 g/kg (locations found in Figure 1). The limits are based upon assessments of microbiological data under a range of environmental conditions—in accordance with the national standard [32].

**Figure 6:**
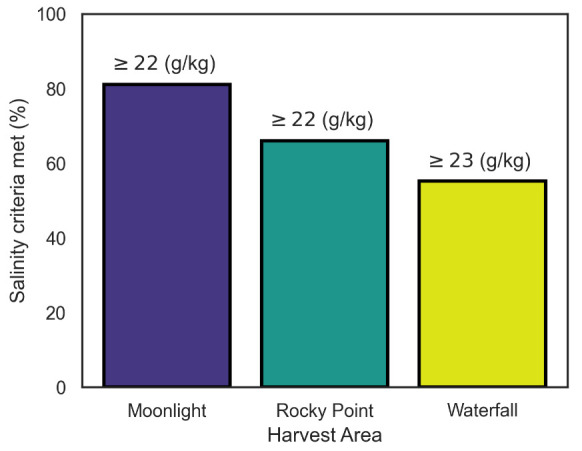
Representation of the percentages of time the harvesting locations have spent in compliance with salinity criteria (see Table A1 for criteria). These percentages were calculated from the average low-tide salinity readings at each harvest area since the network’s deployment. Moonlight and Rocky Point must have salinity readings of ≥22 g/kg, whereas Waterfall’s salinity must be ≥23 g/kg.

Figure 6 illustrates the proportions of time for which the harvest areas have met the salinity criteria from Table A1. Moonlight, which includes Buoys 1, 3, 4 and 5, had the highest harvest open time of 81%. Moving up the river to Rocky Point (Buoys 8 and 9), the percentage of compliance with salinity criteria was only ∼66%. Finally, Waterfall harvesting area (Buoys 10–13) has seen the least compliance with salinity criteria (∼55%). This is to be expected, with saline water being cycled through the river system from Clyde River’s entrance at Batemans Bay (adjacent to Moonlight harvesting area).

The calculated values featured in Figure 6 only represent closures related to salinity; however, the NSW Food Authority, as demonstrated in Table A1, close harvest areas for various reasons, including high *E. coli* concentrations and faecal coliform counts. However, the presence of bacteria such as *E. coli* is generally higher when oysters are situated in low salinity water [33]. Therefore, salinity is suitable for use as a baseline indicator of the state of the river.

### 2.7. Spatial Interpolation

As the sensor network is primarily aimed at increasing oyster farmers’ capacity to make informed farm management decisions, the focus was on presenting these data in an informative and clear manner. Typical two-dimensional representations—such as Figure 5—often fail to portray spatial differences in environmental variables. As these data represent a reasonably complex three-dimensional structure, with ten buoys spatially distributed (spatial-dimension) on fifteen-minute (temporal-dimension) environmental sampling intervals (environmental-dimension), a Python (Version 3.9.5) program was created to formulate heat maps which encompass all these variables [34]. This script uses either *n* data points or a date range of values captured from all buoys to create an array of maps which are then transformed into a graphical interchange file (gif).

Each heat map consists of a spatially interpolated image of salinity (though any environmental variable could be substituted here) readings from all buoys operating on Clyde River with an overlay image captured from OpenStreetMap to visualise the river’s shape [35]. Spatial interpolation is commonly used to infer environmental variables across a water body [36]. The resolution of the spatial interpolation was set to 100 (i.e., 100 values between the maximum and minimum latitude and longitude), as this value provided a balance between computation time and visual aesthetics. As this method cannot interpolate outside of the bounds of the buoys, maximum and minimum latitude and longitude, two *virtual* data points were used for the desired mapping extent with values related to the closest buoys’ *actual* readings (i.e., Buoy 13 for a *virtual* reading in the top left of the map and Buoy 1 for the bottom right).

**Figure 7:**
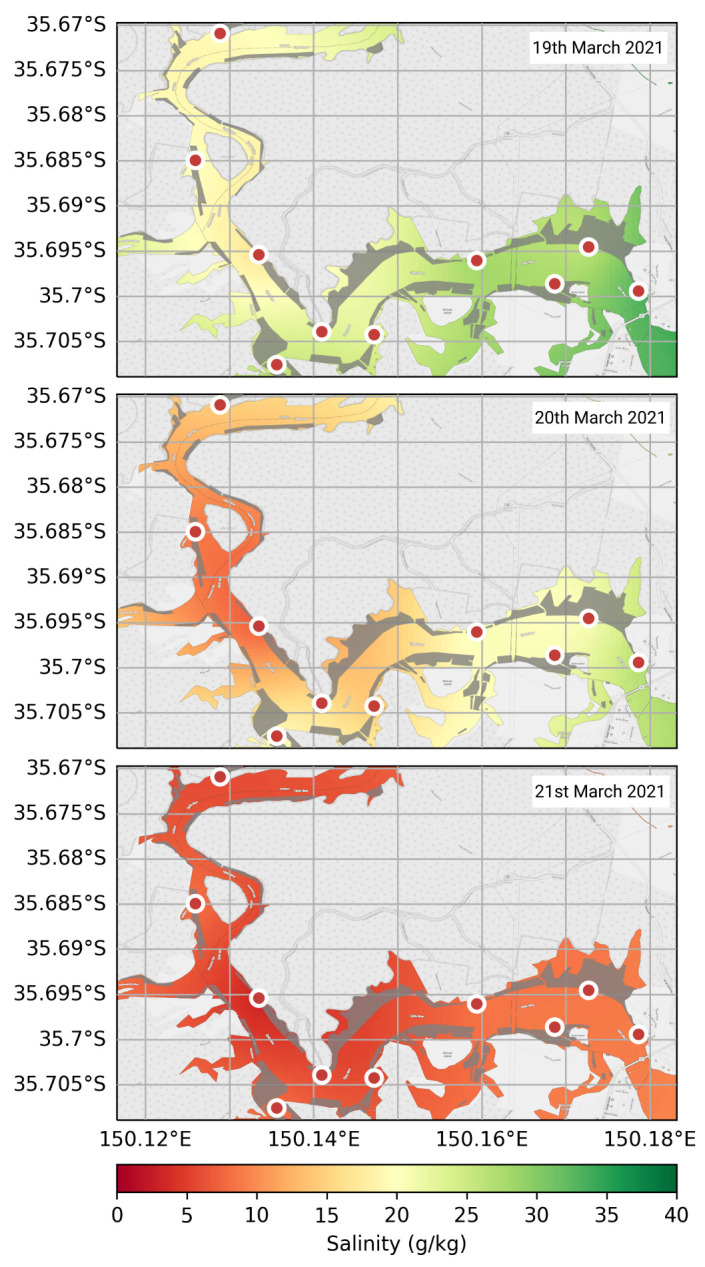
Heat map view of salinity in Clyde River. Colour is used to represent salinity from 0 to 40 g/kg (red and green, respectively). Buoys are represented as red circles with white edges (see Figure 1 for labels). Each consecutive map (from top) represents a 24 h increase in time from the 19 to 21 March 2021. This date range demonstrates the change in salinity on Clyde River after a major rainfall event.

The heat maps featured in Figure 7 demonstrate the effects that large rainfall events can have on Clyde River. Three dates were chosen from March 2021 (19, 20 and 21) which represent the collapse in salinity after a significant flooding event. Buoys located further up the river system experienced declines in salinity sooner than those located close to the rivers mouth (at Batemans Bay), represented as a change in colour from green to red. The heat map representing the state of the river on March 20 indicates that salinity was lower around Buoys 11 and 12, which are located at the exit of a major freshwater tributary—Buckenbowra River. For the 21 of March, the entire Clyde River oyster farming region is represented in red, meaning salinity readings of less than ∼5 g/kg.

## 3. Discussion

This work describes the deployment of a LoRaWAN-enabled sensor network for monitoring both water quality and atmospheric conditions on Clyde River located on the southern coast of NSW, Australia. These data are provided in real-time to oyster farmers operating on the river through a self-hosted data platform known as FarmDecisionTECH${}^{®}$, assisting them in making informed farm management decisions and preventing the loss of oyster leases due to extreme weather events—such as flooding and heat waves. The methods shown in this work represent an end-to-end approach of assisting the aquaculture industry through the use of newly available digital technologies. This differs from other approaches which focus on technological aspects of deploying aquaculture monitoring sensors [26,27,37].

The use of LoRaWAN as a means to communicate sensor data from remote buoys located throughout the river system was integral in ensuring that the entire oyster farming region was represented. There were, however, several problems related to packet *loss* that presented issues for data analysis, as gaps in data were common, occurring about 20% of the time over the operating period of the buoy in some instances (Figure 4). This could be solved in post-processing through interpolation; however, this issue may be relegated in the future using message acknowledgement techniques, which have been shown to reduce packet *loss* to as little as 5% [30]. Additionally, issues created from the LoRaWAN nodes operating on different sampling intervals (phases) could be solved by configuring them to measure at set times (12:00, 12:15, etc.).

The sensor network was deployed in collaboration with oyster farmers as a means to increase their use of valuable digital technologies. A major part of this was the deployment of a real-time weather station, as prior to its installation the oyster farmers were dependent on Mouyra Airport for live weather updates (see Figure 1 for location). As demonstrated in Figure 5, Mouyra Airport was ∼5 ${}^{\circ }$C cooler than Clyde River AWS during a heat wave event at the start of 2021. This may be a result of Moruya Airport’s coastal location, which may be cooled by Northeasterly seabreezes that frequent the east coast of NSW during summer months [38]. Clyde River, on the other hand, is shielded by these seabreezes, which may result in higher temperatures. These temperatures have the potential to kill oysters if they are left exposed, resulting in a substantial loss of income [6]. Interestingly, the coupling of surface air temperature and surface water temperature demonstrates that frequent heat waves could result in surface water temperatures above 30 ${}^{\circ }$C (Figure 5). This would have an impact on oyster farmers, as the current method to protect their leases is to ensure all oysters are submerged in the event of a heat wave. However, if water temperatures exceed 30 ${}^{\circ }$C, this method may prove ineffective. This could be exacerbated under a changing climate: extreme weather events, such as heat waves, are predicted to increase in frequency and intensity [39]. During this three month period (Dec–Feb) there were 378 unique page views on the FarmDecisionTECH${}^{®}$ Clyde River dashboard, which represents a substantial number of views considering there are ∼17 growers operating on the river.

In addition to responding to extreme weather events, real-time in situ data can also be used for basic day-to-day decisions. This includes the monitoring of low to moderate intensity rainfall and its effect on particular harvesting areas. These data and representations such as Figure 6 also help with an important aspect of oyster farming—risk management. New oyster farmers could use these data to determine the suitability of particular oyster growing regions, in terms of how often these regions are generally open for harvesting. Existing farmers can use these data to determine where their particularly vulnerable oysters should be distributed amongst their leases.

Communicating environmental information to farmers was also seen as a major priority in the deployment of this infrastructure. Oyster farmers are intrinsically tied to environmental conditions through their daily operations, and to harvesting regulations laid out by NSW Food Authority (Table A1). The typical protocol for closing harvesting areas depends on a number of variables, including, rainfall, bacteria count, faecal coliform count and salinity. The latter is easier to measure in situ and therefore can be provided in real-time through graphical visualisations such as Figure 7. The NSW Food Authority requires physical sampling around mid-ebb tide; however, sensor networks, such as the one described in this work, could be adopted as an official method of assessing the state of the river—subject to approval. These in situ measurements could speed up these processes and allow oyster farmers to resume harvesting soon after the river has recovered.

While Figure 7 provides a clear and informative view on the state of Clyde River for oyster farmers, there are several problems with this representation that need to be addressed. (1) The map does not represent currents and eddies that may be influencing the river. (2) The interpolation does not occur outside the bounds of the buoys’ spatial locations; therefore, *virtual* data points are placed to delimit the desired map size, which use data from the closest buoys. (3) This interpolation only represents the upper ∼40 cm of water; however, this region is where oysters are located. (4) The environmental measurements must have sufficient spatial resolution. While these problems exist, our heat maps still provide valuable rapid representations of the state of a river for oyster farmers. The example shown in Figure 7 demonstrates the change in salinity in Clyde River after a major rainfall event. Two major tributaries include Buckenbowra River (entering Clyde River around Buoy 11), and ∼30 km further up, Clyde River at Brooman. As can be seen from Figure 7, on the 20 March 2021, Buoys 11 and 12 were lower in salinity than Buoy 13, indicating that significant flow was occurring from Buckenbowra River and that flow from upstream tributaries had not reached Buoy 13. The following day (21 March), the entire Clyde River was inundated with freshwater, resulting in a decline in salinity. The use of in situ sensors does allow oyster farmers the ability to monitor the river’s state and can provide a quantitative assessment on its subsequent recovery so farming operations can return to normal.

## 4. Conclusions

This work has described the deployment of a real-time sensor network to monitor environmental conditions on Clyde River. Data captured from this network have been used to enhance farm management decisions of oyster farmers operating on the river. The use of relatively low-cost in situ sensors has proven to be an essential asset to ensure oyster farmers have both current and accurate information with which to make decisions. Undoubtedly, the use of LoRaWAN and other IoT systems has made the deployment of this sensor network possible. Other oyster farming regions could benefit from similar infrastructure, and it could provide valuable information needed to help farmers adapt to the changing climate.

To increase the ability for oyster farmers to make informed farm management decisions, subsequent works could focus on predicting future environmental conditions. This would allow farmers to prepare for flooding or heat wave events and take preventive measures before the onset of such conditions. Additionally, the sensors deployed in this work only provide basic environmental variables, such as temperature and salinity. However, as mentioned throughout this work, other factors, such as phytoplankton blooms, bacteria and coliform faecal count, play large roles in how oyster farmers operate. This presents opportunities for new in situ environmental sensors that can assess these variables in real-time [40,41].

## Abbreviations

The following abbreviations are used in this manuscript:

API	Application programming interface
AWS	Automatic weather station
BoM	Bureau of Meteorology
FCC	Faecal coliform count
GIF	Graphical interchange file
IALA	International Association of Marine Aids to Navigation and Lighthouse Authorities
IoT	Internet of Things
JSON	Java script object notation
LPWAN	Low power wide area network
LoRaWAN	Low power, long range, wireless area network
MQTT	Message queuing telemetry transport
NB-IoT	Narrow-band Internet of Things
NSW	New South Wales
RSSI	Received signal strength indicator
SNR	Signal-to-noise ratio
TTN	The Things Network

## Appendix A

**Table A1:** NSW Food Authority (unpublished) operational criteria for harvest area management on Clyde River.

Harvest Zone	24 h Rainfall (mm) *	7 day Rainfall (mm)	FCC (cfu/100 mL)	Bacteria (*E. coli*/g)	Salinity (g/kg)
Moonlight	30	100	≤14 or ≤70 **	≤2.3 or ≤46 **	≥22 or >18 **
Rocky Point	40	100	≤14	≤2.3	≥22
Waterfall	40	100	≤14	≤2.3	≥23

* Rainfall must be ≤10% of weekly closure limit after sampling to open harvesting. ** Restricted opening, oysters must undergo depuration in high quality water to purge contaminants. Note: Opening of harvesting areas also depends on other environmental assessments, such as phytoplankton sampling and identification.
